# ACT FOR OLDER VETERANS: TELEHEALTH-BASED ACCEPTANCE AND COMMITMENT GROUP THERAPY FOR OLDER VETERANS

**DOI:** 10.1093/geroni/igae098.3425

**Published:** 2024-12-31

**Authors:** Johnna Kelley, Deanna Dragan, Katherine Luci

**Affiliations:** Salem VA Healthcare System, Salem, Virginia, United States; Salem VA Healthcare System, Salem, Virginia, United States; Salem VA Healthcare System, Salem, Virginia, United States

## Abstract

Acceptance and Commitment Therapy (ACT) is a transdiagnostic intervention with promise for use with older adults due to its relevance to a variety of mental health conditions, overall well-being, and adjustment to life changes. This pilot study (n = 10) sought to demonstrate that a novel group therapy intervention for older adults, delivered via telehealth, is efficacious and acceptable to Veterans aged 60 years of age or older. Retention rates (10/10) and attendance (M = 12.7/16 sessions) of study completers suggest feasibility and acceptability of the group. Six participants demonstrated high engagement (attended ³ 12 sessions) and four demonstrated average engagement (attended 5-11 sessions). Participants completed a structured, post-intervention interview; feedback was analyzed for themes and generally supported acceptability of the intervention. Additional themes referred to participants’ experience of the virtual format, connections made with other group members, and areas for improvement. Finally, paired sample t-tests were conducted to analyze pre-post measures of anxiety, depression, loneliness, well-being, and engagement in meaningful activities. Outcome data suggests no statistically significant changes on these measures; however, the decrease in scores on a measure of depression reflects a small to moderate effect size (t(9) = 1.467, p =.18, d =.46). Future studies with larger sample sizes are needed to determine the impact of treatment engagement on these outcome measures. Overall, preliminary outcome data combined with qualitative feedback from participants, retention rates, and attendance rates, suggest that ACT delivered in a virtual group format is acceptable, feasible, and likely beneficial to older Veterans.

